# Intratubular crystal formation in the exposed dentin from nano-sized calcium silicate for dentin hypersensitivity treatment

**DOI:** 10.1038/s41598-023-30351-2

**Published:** 2023-08-30

**Authors:** Mi-Jeong Jeon, Jeong-Won Park, Deog-Gyu Seo

**Affiliations:** 1 Department of Conservative Dentistry, School of Dentistry and Dental Research Institute, Seoul National University, Seoul, Republic of Korea; 2 Department of Conservative Dentistry, College of Dentistry, Gangnam Severance Hospital, Yonsei University, Seoul, Republic of Korea

**Keywords:** Dental diseases, Biomineralization

## Abstract

The aim of this study is to evaluate intratubular crystal formation from the experimental material consisting of dicalcium silicate (C_2_S) and tricalcium silicate (C_3_S) with nano-scaled particle size. A total of twenty-four specimens were made by isolating 8 mm of the cervical part centered at the cementoenamel junction of extracted premolars. Twelve specimens were not treated and considered as control. The experimental material was applied to the other twelve specimens by brushing for 10,000 strokes. Each group was randomly divided into four subgroups according to the period of immersion in phosphate buffer saline (PBS) for 1, 30, 60, and 90 days each. The specimens were sectioned longitudinally and examined with scanning electron microscopy and energy dispersion X-ray spectroscopy. The intratubular crystal were formed in PBS and densely filled the dentinal tubules over time. The crystal formation occurred at a depth of more than 50 μm from the dentin surface. The Ca/P ratio of formed intratubular crystals was 1.68 after 3 months. The experimental material consisting of C_2_S and C_3_S with a nanoscale particle size can form hydroxyapatite-like crystals in dentinal tubules in PBS, and there is a possibility of reducing dentin hypersensitivity by blocking the dentinal fluid flow.

## Introduction

Dentin hypersensitivity (DH) is defined as “pain arising from exposed dentin in response to stimuli, typically thermal, evaporative, tactile, osmotic or chemical, which cannot be ascribed to any other form of dental defect or pathology”^1^. According to hydrodynamic theory, teeth with dentin hypersensitivity have opened dentinal tubules with pulpal patency^2^, a channel for stimulation transmission by moving fluids within the tubules^3^.

There are several approaches to reduce discomfort from DH. Potassium nitrate could decrease the excitability of the intradental nerve fibers and achieve pain relief^4^. Although the effect of relief dentin hypersensitivity appears immediately, it does not last long^5^, and it is no substantial evidence supporting its efficacy^6^. Another approach is occluding the tubules by hindering the tubular fluid movement. The concept of tubule occlusion is a logical extension of the hydrodynamic theory^7^. Products including strontium, bioactive glass particles, amorphous calcium phosphate, arginine, oxalate, and fluoride can be found on the market using ions and salt. The limitations of these desensitizers are related to lack of intratubular occlusion due to large particle size and high solubility of the occluding materials, and short duration of effective time due to poor resistance to acid attacks^8–10^. Moreover, the desensitizing effects depend significantly on the individual’s sensory threshold^11^.

Effective occlusion of dentinal tubules is difficult due to the small dimensions of dentinal tubules (0.5–4 μm in diameter)^2^, the complex structure of a pulp-dentin system, the outward hydraulic pressure of dental pulp (0.15 kg/cm^2^)^12^. For effective management of dentine hypersensitivity, new materials are required to penetrate deep enough into dentinal tubules, last a long time, and not easily wash out with the oral environment^13^. Various types of materials, such as bioceramic, synthetic polymer, and peptides, were attempted as an alternative desensitizer. However, these alternative materials did not completely overcome the disadvantages of the existing desensitizers mentioned above^14, 15^.

Calcium silicate, can form calcium phosphate precipitation when contacted with physiological fluid containing phosphate^16, 17^. A tag-like structure in the dentinal tubule was observed after calcium silicate based sealer application during the root canal filling procedure^18^. However, no studies have used calcium silicate as a desensitizer and applied it to outer dentin surface over a period time.

The aim of this study was to evaluate that intratubular crystals were formed in dentinal tubules when experimental material consisting of dicalcium silicate (2CaO⋅SiO_2_, C_2_S) and tricalcium silicate (3CaO⋅SiO_2_, C_3_S) was applied to the exposed outer dentin surface under PBS for 3 months.

## Results

**Figure 1:**
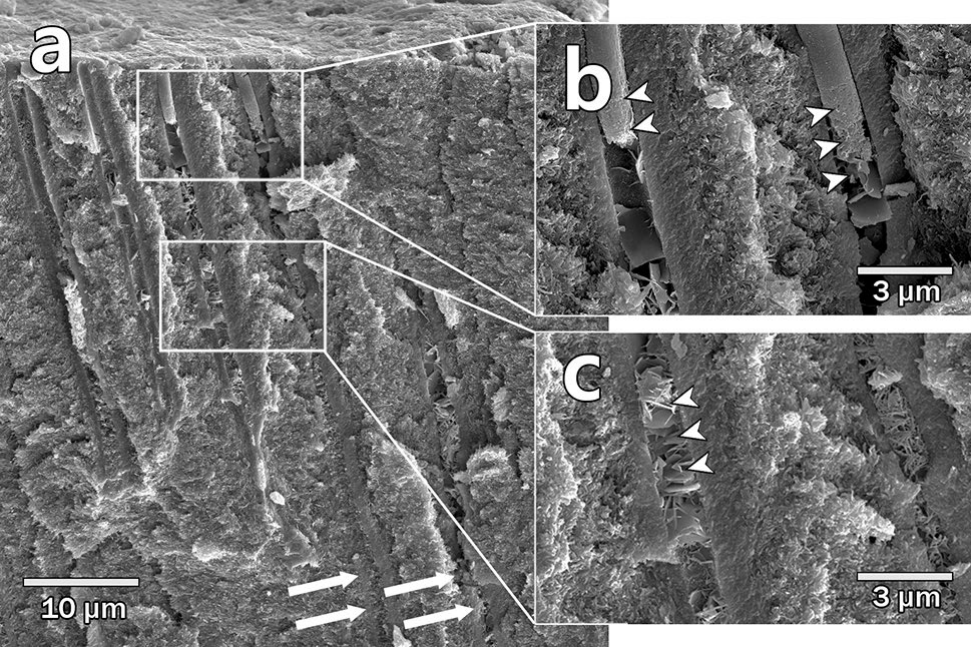
SEM images of longitudinally sectioned specimens immersed for 2 months in PBS. (**a**) The white arrows show that the intratubular crystals were formed to a depth of more than 50 μm from the exposed dentin surface. (**b**) Plug-shaped precipitate below dentin surface (white arrowheads). (**c**) plate-shaped crystals in the dentinal tubule (white arrowheads).

Figure 1 represented SEM images of a longitudinally sectioned specimen immersed for 2 months in PBS after applying the experimental material consisting of C_2_S and C_3_S. There were plug shape precipitates below the dentinal tubule orifice. The lower surface of the occluding plug became rough, and plate-shaped intratubular crystals were formed below the occluding plug. The intratubular crystals were formed at a depth of more than 50 μm from the surface of the exposed dentin surface (Fig. 1 white arrows).

**Figure 2:**
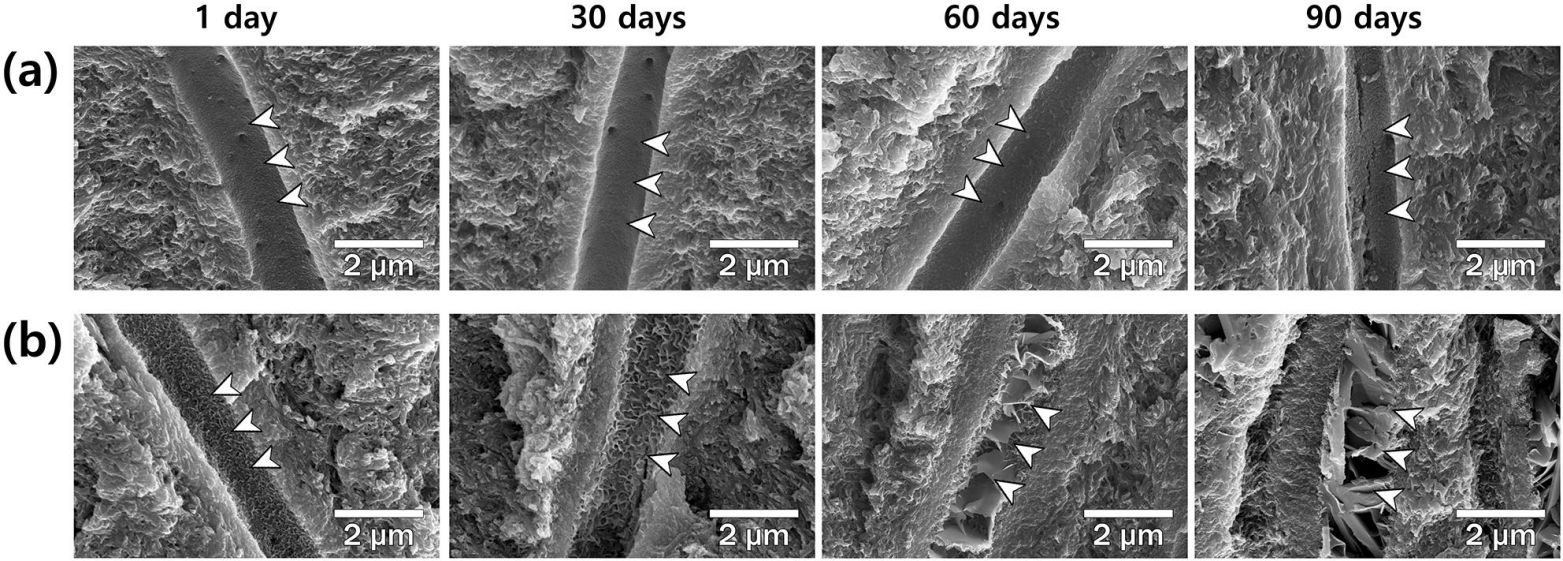
SEM images of longitudinally sectioned specimens (× 50,000). (**a**) Control group and (**b**) C_2_S/C_3_S group of 1, 30, 60, and 90 days after immersion in PBS, respectively. For the entire observation period, no intratubular crystal formation in the control group (**a**, white arrowheads). Crystal formation was observed in dentinal tubules forming a denser crystal complex as the period of storage in PBS increased (**b**, white arrowheads).

Figure 2 shows SEM images of longitudinally sectioned specimens that illustrate the dentinal tubules within a range of 20–100 μm from the dentin surface. There was no intratubular crystal formation in all specimens for the entire observation period in the control group (white arrows in a). In the experimental group, the roughening of the inner surface of the dentinal tubules was observed after 1 day, and plate-shaped crystals were formed in the dentinal tubules after 2 months. As the period stored in the media gets longer, the plate-shaped crystals are linked together to form a denser collection (white arrows in b).

The composition of the intratubular crystals in PBS was evaluated using EDS (Table 1). After immersion in PBS for 1 day, the Ca/P ratio of the crystal was 1.57. Then, the Ca/P ratio increased to 1.68 after 90 days, which meant the crystals from C_2_S/C_3_S in PBS were supposed to be the hydroxyapatite-like crystal, of which the Ca/P ratio was 1.67.

**Table 1:** Ca/P ratio of the intratubular crystals.

Time	1 day	30 days	60 days	90 days
Ca/P	1.57	1.59	1.64	1.68

## Discussion

According to hydrodynamic theory, blocking the opened dentinal tubules could reduce discomfort from dentin hypersensitivity by reducing fluid movement through dentinal tubules^3^. In this study, the intratubular crystals were formed in PBS after the experimental material was applied to the exposed dentin surface and filled the dentinal tubules more densely as the period of storage in PBS increased.

In the case of intratubular crystal formation in previous study^18^, when using a calcium silicate based sealer for root canal treatment, calcium silicate was continuously present inside the root canal and in contact with the dentinal tubules. For dentin hypersensitivity treatment, a desensitizer should be applied to the dentin surface exposed to the outside, which is not in contact with the surface of dentin continuously exposed due to physical and chemical factors in the oral cavity. Therefore, in order to improve intratubular occlusion, desensitizer must efficiently penetrate the dentinal tubules.

In this study, two efforts were made to achieve efficient penetration of experimental particles as greater penetration of particles into the dentinal tubules would have occurred. First, the experimental used in this study consisted of particles with a smaller diameter than in other studies using calcium silicate (lower than 85 μm or 0.5 mm)^19, 20^ or calcium silicate in commercially available mineral trioxide aggregate (MTA) products ^21^. The diameter of dentinal tubules of the sensitive dentin is larger than that of the non-sensitive dentin but is only 0.83 μm on average^2^. The small dimension of dentinal tubules makes it difficult for desensitizers to be efficiently penetrated^12^. According to a study by Kim et al.^22^, comparing the degree of absorption according to the particle size of the desensitizer, the desensitizer with small-sized particles had more opportunities to penetrate the dental tubules. Therefore, the experimental materials, which consist of specially designed particles with sizes ranging from tens to hundreds of nanometers (as depicted in Fig. 4, with particle sizes ranging from 0.052 μm (D0.1) to 1.267 μm (D0.9)), are likely to effectively penetrate the dentinal tubules and form occluding plugs, as demonstrated in previous studies^23^. Second, C_2_S/C_3_S were applied as a brushing motion to the dentin surface. This simulated that calcium silicate was contained in toothpaste and applied to the surface of the dentin when brushing. A total of 10,000 repeated strokes (1 strokes/s) of brushing simulated about 18.5 days assuming brushing 3 min/time and three times a day^24^. It can have a chance to push desensitizing materials into dentinal tubules compared with just dropping or rubbing with a micro-brush on the specimen surface. As a result, the occluding plug was formed below the dentinal tubule orifice in applying experimental material to the brushing motion (Fig. 1).

The occluding plugs can act as a reservoir of calcium ions, continuously dissolving calcium ions and making the inside of the dentinal tubules into a supersaturation state^25^. The supersaturation condition was expected to cause local aggregation of calcium ions and phosphate ions due to interaction with ions present on the inner surface of the dentinal tubules, which caused the growth of the intratubular crystals^23^. This reaction can be inferred that the lower part of the occluding plug was rough, and plate-shaped crystals were formed below (Fig. 1b white arrowheads).

The intratubular crystal formation reaction by diffusion according to the concentration gradient of ions can occur at a deep point in the dentinal tubules. In this study, the intratubular crystals were formed at a depth of more than 50 μm from the exposed dentin surface (Fig. 1a white arrows). In a clinical situation, superficial occlusion of dentinal tubules has a short-term effect as the precipitates can be easily removed due to daily tooth brushing, dissolution by saliva, and acidic beverages^26^. For the long-term effect of desensitizer, the material blocking the dentinal tubules should be deep enough^27^.

Crystal formation reaction continuously takes place in dentinal tubules forming denser crystal complex as the period of storage in PBS increased (Fig. 2). The crystal complex is expected to contribute to preventing the movement of the pulpal fluid through the dentinal tubules and reduce discomfort from dentin hypersensitivity more effectively^7, 28^. Further research will be needed on the clinical effectiveness of the experimental material.

When C_2_S and C_3_S are in contact with water, they are hydroxylated, and the surface dissolves according to below^29^;

1 $$2(2{\text{CaO}} \cdot {\text{SiO}}_{2} ) + 4{\text{H}}_{2} {\text{O}} \to 3{\text{CaO}} \cdot 2{\text{SiO}}_{2} \cdot 3{\text{H}}_{2} {\text{O}} + {\text{Ca}}\left( {{\text{OH}}} \right)_{2}$$

2 $$2(3{\text{CaO}} \cdot {\text{SiO}}_{2} ) + 6{\text{H}}_{2} {\text{O}} \to 3{\text{CaO}} \cdot 2{\text{SiO}}_{2} \cdot 3{\text{H}}_{2} {\text{O}} + 3{\text{Ca}}\left( {{\text{OH}}} \right)_{2}$$

As a result of these reactions, calcium and hydroxyl ions are released, resulting in a highly alkaline environment^30^. After the hydration reaction of calcium silicate, the hydroxyapatite-like crystals were formed under contact with PBS^31, 32^. This reaction results in the formation of hydroxyapatite through a continuous process of calcium dissolution from the experimental material and the continuous provision of phosphate ions from the PBS. A previous study showed calcium-deficient and B-type carbonated apatite with a 1.4–1.5 Ca/P ratio formed from Portland cement in PBS^32^. Other studies showed calcium silicate in PBS could make hydroxyapatites after hydration^25, 33, 34^. In this study, the Ca/P ratio of formed intratubular crystals was 1.68 after 3 months. Considering that the Ca/P ratio of hydroxyapatite is 1.67^35^, it can be expected that the intratubular crystals made in this experiment would be hydroxyapatite-like crystals. The Ca/P ratio is a measure of the mineralization of the formed crystal, and an increase in this ratio over time indicates that the intratubular crystal is progressing towards higher levels of mineralization.

However, this experimental design could not simulate an oral environment, such as an acid challenge from the diet. Therefore, further studies need to be performed to evaluate the effect of the acid-neutral cycle on intratubular crystal formation.

This study demonstrated that the experimental material could form intratubular crystals in PBS after being applied to the exposed dentin surface. The crystal formation occurred at more than 50 μm from the dentin surface, and the crystals more densely filled the dentinal tubules over time. The experimental material consisting of C_2_S and C_3_S with nano-scaled particle size used in this study allowed for effective penetration to dentinal tubules and the formation of intratubular crystals, which makes the material a promising alternative for clinical use to reduce discomfort from dentin hypersensitivity.

## Methods

### Specimen preparation

This study was approved by the Ethics Committee of the Seoul National University, Graduate School of Dentistry (IRB number: S-D20190010). Total 24 recently extracted human premolars for orthodontic treatment with intact coronal and root surfaces were prepared. They were stored in 0.1% thymol solution to inhibit microbial growth for no longer than 3 months prior to use. Debris on the surface of all teeth was removed by perio-curette and examined whether there were crack lines under a microscope (200×) (Carl Zeiss Surgical GmbH, Oberkochen, Germany).

**Figure 3:**
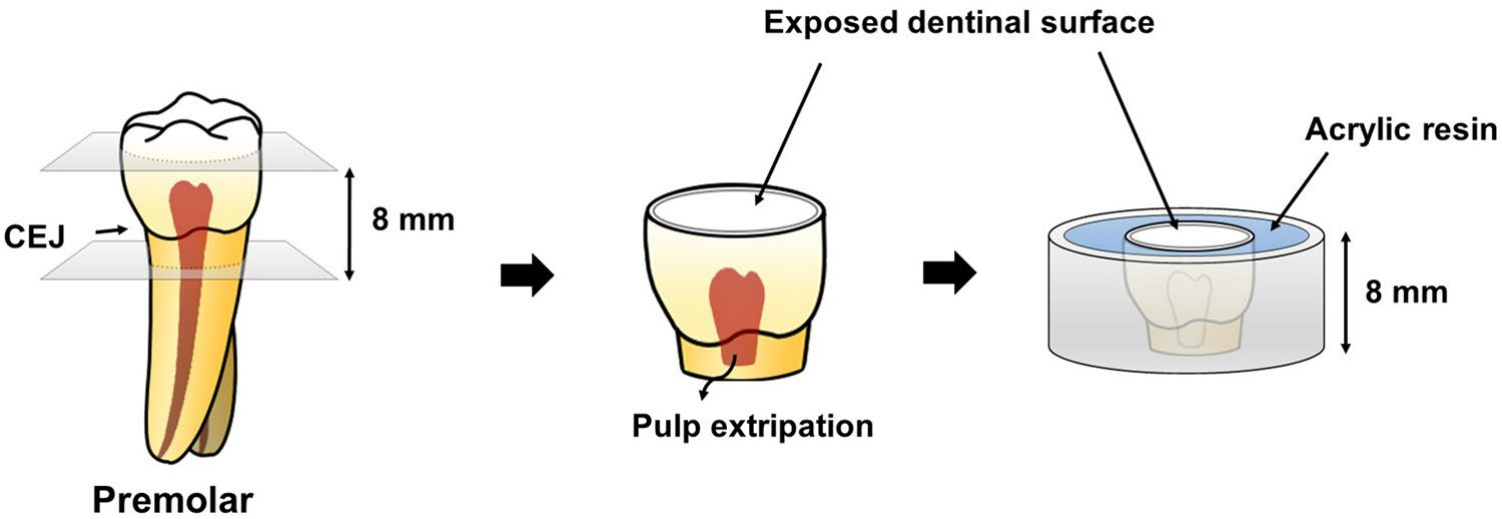
Schematic illustration of specimen preparation. Total 8 mm of cervical part centered at the cementoenamel junction was isolated. Remained pulp tissue was removed and sectioned tooth was mounted in the ring-shape acrylic mold.

A specimen with a total length of 8 mm was made by cutting the 4 mm above and below the cemento-enamel junction with a low-speed diamond saw (Isomet™, Buehler, Lake Bluff, IL, USA) under constant water cooling. Flat and fresh dentin was exposed on the occlusal side surface without perforation at the pulp horn area. Remained pulp tissue was removed carefully with small forceps without touching the inner part of the pulpal space. Then, the sectioned tooth was mounted in the ring-shaped acrylic mold with self-cured acrylic resin (Bosworth Fastray, Keystone Industries GmbH, Singen, Germany) (Fig. 3).

The exposed upper dentin surface of each specimen was treated with 17% ethylenediaminetetraacetic acid (EDTA) for 1 min, followed by 2.5 mL of 5.25% sodium hypochlorite to open dentinal tubules and remove the smear layer on the exposed dentin surface and rinsed with 10 mL of distilled water twice.

### C_2_S/C_3_S preparation and application

**Figure 4:**
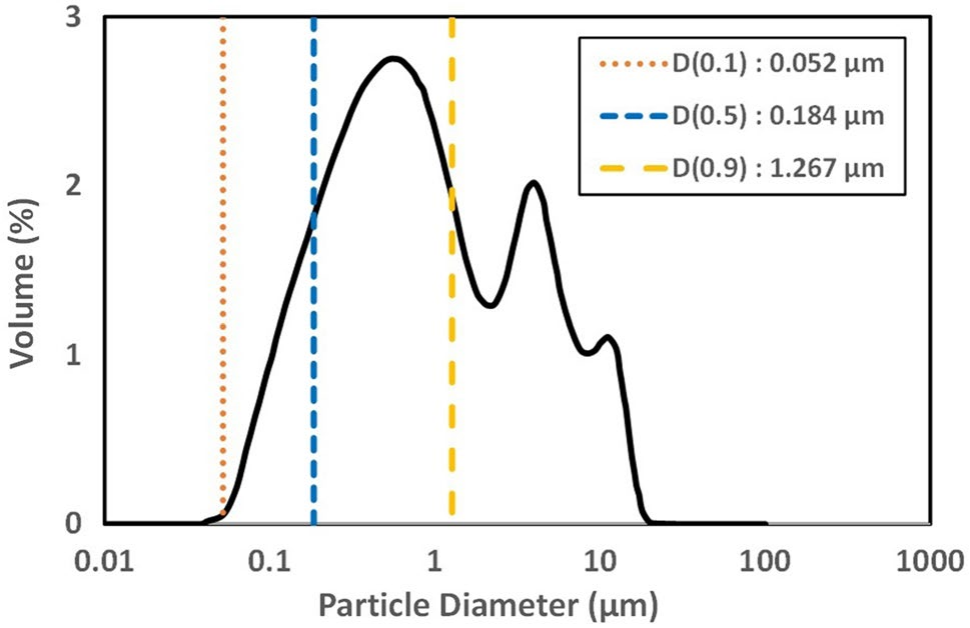
Particle distribution in diameter of experimental material (C_2_S/C_3_S). The particle size of the experimental material was distributed between 0.052 μm (D0.1) and 1.267 μm (D0.9), and the median value (D0.5) is 0.184 μm.

The experimental material consisting of dicalcium silicate and tricalcium silicate (C_2_S/C_3_S) was prepared by a sol–gel method as Zhao and Chang^36^ using CaCO_3_, SiO_2_, Al_2_O_3_ as the raw materials and the obtained material calcined at 1450 ℃ for 6 h. The resultant powders were ground at 300 rpm using a Disk Mill (Disk Mill, KM tech, Icheon, Republic of Korea) and then at 200 rpm using a Ball Mill (BML-2, DAITHAN SCIENTIFIC GROUP, Wonju-si, Republic of Korea) for 48 h. Table 2 shows the main component and content of the experimental material. The particle size of the experimental material was distributed between 0.052 μm (D0.1) and 1.267 μm (D0.9), and the median value (D0.5) is 0.184 μm (Fig. 4).

**Table 2:** Experimental material of the study.

Groups (n = 12)	Component	Content (wt%)
C_2_S/C_3_S	Dicalcium silicate (2CaO⋅SiO_2_, C_2_S)	14.91
	Tricalcium silicate (3CaO⋅SiO_2_, C_3_S)	74.21
	Others Tri-calcium aluminate (3CaO⋅Al_2_O_3_, C_3_A) Tetra-calcium alumino ferrite (4CaO⋅Al_2_O_3_⋅Fe_2_O_3_, C_4_AF) Periclase Lime	10.88

After preparation of the specimens, 0.5 g of C_2_S/C_3_S was mixed with 5 mL of distilled water and applied on the exposed dentin surface of the specimen by tooth brushing motion according to ISO 11,609 standards for dentin wear test, abrasive in the dentifrice is about 10% of dentifrice and water mixture. The concentration of tricalcium silicate applied is followed this standard. A total of 10,000 repeated strokes (1 strokes/s) were applied using the toothbrush onto each specimen under a 150 g-load continuously being touched among test material mixtures and the exposed dentin surface.

The specimens were randomly divided into four subgroups according to the period of immersion in PBS (D8662, Sigma-Aldrich, St. Louis, MO, USA) media for 1, 30, 60, and 90 days each at 37 ℃ (n = 3). The PBS solution was replaced every 7 days. The composition (in g/L) of used PBS was CaCl_2_⋅2H_2_O 0.133, MgCl_2_⋅6H_2_O 0.1, KCI 0.2, KH_2_PO_4_ 0.2, NaCl 8.0, Na_2_HPO_4_ (anhydrous) 1.15, and the pH was 7.4.

### Scanning Electron Microscope analysis and EDS analysis

The specimens were longitudinally sectioned, and six sectioned surfaces in each group were examined to assess crystal formation in dentinal tubules after experimental material application on exposed upper dentin surfaces. All specimens were mounted on aluminum stubs and sputter-coated with a 30 nm layer of gold and examined using field emission scanning electron microscopy (FE-SEM, Apreo S; Thermo Fisher SCIENTIFIC, Waltham, MA, U.S.A.). The intratubular crystals were examined by energy dispersive spectroscopy (EDS, XFlash 6160, Bruker, Germany) to analyze the components.

### Ethics declarations

This study was approved by the Ethics Committee of the Seoul National University, Graduate School of Dentistry (IRB number: S-D20190010). All biological samples were included after obtaining the informed consent from all subjects. All methods were conducted in accordance with Declarations of Helsinki.

## Data Availability

The data that support the findings of this study are available from the corresponding author upon reasonable request.
